# Source Fingerprinting of PFOA via Full- and Intramolecular Stable Isotope Ratios of Carbon Using Orbitrap-IRMS

**DOI:** 10.1021/acs.analchem.5c06168

**Published:** 2025-12-13

**Authors:** Holden M. Nelson, Zhiliang Xu, Hui Li, James J. Moran

**Affiliations:** † Department of Integrative Biology, Michigan State University, East Lansing, Michigan 48824, United States; ‡ Center for PFAS Research, 3078 Michigan State University, East Lansing, Michigan 48824, United States; § Department of Chemistry, Michigan State University, East Lansing, Michigan 48824, United States; ∥ Department of Plant, Soil, and Microbial Sciences, Michigan State University, East Lansing, Michigan 48824, United States; ⊥ Ecology, Evolution, and Behavior Program, Michigan State University, East Lansing, Michigan 48824, United States

## Abstract

Remediation approaches for reducing environmental loading of perfluoroalkyl and polyfluoroalkyl substances (PFAS) require the ability to track PFAS origin sources. One common source attribution method for environmental contaminants is stable isotope analysis, with magnetic sector isotope ratio mass spectrometry (IRMS) being the workhorse for making these measurements. However, relatively large sample requirements and the requisite deconstruction of analytes to simple gases (e.g., CO_2_) inhibit the application of IRMS to PFAS in environmental contexts, where even trace PFAS concentrations can exhibit toxicological effects, and decomposition of PFAS yields instrument-degrading HF. To circumvent these limitations, we developed an electrospray ionization Orbitrap-IRMS method to determine stable carbon isotope (^13^C) ratios of PFAS with reduced sample quantities, without producing HF, and with the additional capability of performing intramolecular isotope ratio measurements for increased sample discrimination potential. We demonstrate this approach using perfluorooctanoic acid (PFOA) as a model compound. We collected twenty-three samples of neat, commercially available PFOA with different lot numbers from 15 different suppliers and determined that the full molecule stable carbon isotope ratios span ∼25‰ with a measurement precision of 0.7‰. The stable isotope ratios of the decarboxylated fragment of PFOA span ∼28‰ with a measurement precision of 0.2‰. Of the 253 possible pairwise comparisons between the samples, 230 are significantly different based on the combined full and intramolecular isotopic signatures. The isotopic analyses suggest a correlation with the PFOA synthesis method. Our results highlight the potential of Orbitrap-IRMS as a forensic tool for the source attribution of PFAS contaminants.

## Graphical abstract

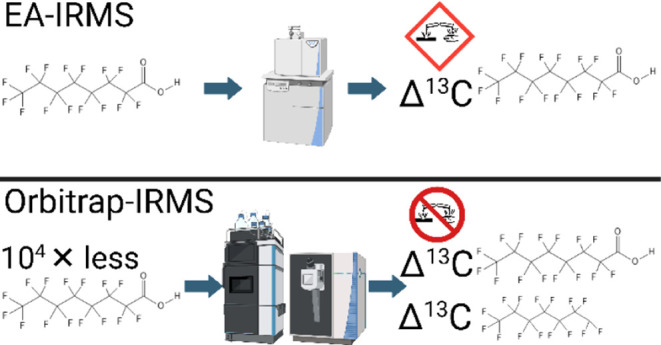

## 1 Introduction

Per- and polyfluoroalkyl substances (PFAS) make up a broad class of synthetic organofluorine compounds that have been mass-produced since the 1950s. As surfactants with high thermal and chemical stability, PFAS are incorporated into a wide range of industrial and consumer products, including fire-fighting foams, nonstick cookware, and fast-food packaging, among many others.^1–6^ Their prevalence in an expansive array of goods and industrial processes has led to the near-ubiquitous occurrence of PFAS in ecosystems around the globe.^7–12^ This, combined with documented and predicted health consequences from even trace PFAS exposure, has propelled PFAS to the forefront of many environmental efforts, with a focus on reducing PFAS exposure and developing remediation strategies for cleaning up PFAS contamination.^13–15^ Gauging the success of remedial efforts requires tools for monitoring PFAS provenance and transport in the environment. Current environmental forensic methods for PFAS contamination primarily rely on the use of chemical and isomeric fingerprints.^16^ Standard methodologies for testing PFAS concentrations only detect a limited set of congeners, and the prevalence of a limited number of different congeners across multiple industries can result in suspected sources sharing a chemical signature.^17^ Major synthesis pathways of PFAS compounds generate isomerically distinct products, with fluorotelomerization producing linear-only isomers, whereas other methods, such as electrochemical fluorination (ECF) and liquid-phase direct fluorination (LPDF), generate some branched isomers in addition to the primarily linear product.^18^ However, the resolving power of isomer ratios can only be used to distinguish between the two different synthesis pathways, and there is currently not sufficient evidence to suggest that two distinct sources of PFAS compounds produced by the same method can be reliably differentiated by this method alone.^19^

Stable isotope ratios can provide critical source attribution insights for environmental pollutants, including hexachlorocyclohexanes,^20^ organophosphorus pesticides,^21^ and other halogenated organic compounds.^22^ Variations in intrinsic stable isotope ratios can be imparted by the method utilized to synthesize a compound and differences in the isotope ratios of precursors, providing complementary potential to discriminate the origin of pollutants beyond what chemical and isomeric content can provide alone. While magnetic sector isotope ratio mass spectrometry (IRMS) is the traditional workhorse for stable isotope measurements, there are two key limitations to using IRMS for PFAS analysis. First, elemental analyzer IRMS measurements of common PFAS congeners require ∼1 mg of analyte per analysis,^23^ an unrealistically high quantity in environmental samples where even trace amounts of PFAS pose an environmental concern. Second, IRMS requires complete conversion of the analyte into a simple gas prior to ionization—a challenge for PFAS compounds due to both the stability of the carbon–fluorine bonds and the resultant HF produced, which can damage the instrument.

Although IRMS-based stable isotope analysis of PFAS may be untenable, emerging techniques in high-resolution mass spectrometry offer an alternative approach.^24^ The ability of Orbitrap-IRMS to measure isotope ratios in inorganic and organic compounds has recently been demonstrated across a variety of applications.^24–27^ In comparison to IRMS, Orbitrap requires smaller sample sizes (pico- to nanomoles versus micromoles C) per analysis. Also, Orbitrap-IRMS measurements can be performed using electrospray ionization (ESI)^28^—circumventing the otherwise requisite combustion/oxidation step. Further, Orbitrap-IRMS allows for measurements of intramolecular isotope ratios via analysis of ion fragments generated in the ESI source.^27^ The implementation of intramolecular stable isotope analysis has historically been reliant on the use of site-specific natural isotopic fractionation nuclear magnetic resonance (SNIF-NMR)^29^ and pyrolysis gas chromatography IRMS (py-GC-IRMS).^30^ Recent studies have shown great potential with NMR-based isotopic analyses of organofluorines.^31^ However, like magnetic sector IRMS, SNIF-NMR requires significantly more sample than is feasible for most field sampling of PFAS compounds, with analyses typically requiring milligrams of analyte per measurement. Pyrolytic techniques, much like other chemical processes to convert analytes to simple gases, produce HF as a reaction byproduct, making it impractical for intramolecular isotopic analyses of PFAS.

Here, we sought to test whether Orbitrap-IRMS could be used to obtain high-precision stable isotope measurements of PFAS compounds, using perfluorooctanoic acid (PFOA) as a model compound due to its widespread presence at contaminated sites and prominence in the literature.^32^ We wanted to further evaluate whether PFOA contains sufficient variation in stable isotopic content to make this a useful intrinsic signature for advancing environmental fingerprinting and source attribution efforts. We describe methodological developments used for stable isotope measurements of both PFOA and a decarboxylated fragment of PFOA, and discuss the ability to use stable isotope analysis to fingerprint these samples.

## 2 Methods

### 2.1 Materials and Sample Information

We collected twenty-three samples of PFOA from multiple suppliers with differing lot numbers. We prepared 1.0 mg L^–1^ (2.4 μM) working solutions in 50:50 (v:v) LC-MS grade acetonitrile (Fisher Chemical) and LC-MS grade methanol (Fisher Chemical) with 0.1% ammonium acetate (Thermo Scientific Chemical) for isotopic analyses. Samples diluted to 10 μg L^–1^ (24 nM) PFOA were prepared from the working solutions of PFOA in methanol for isomeric analyses. Sample information is provided in Table S.A.1. PFOA-01 was used both as a sample and as a reference solution.

### 2.2 UHPLC-Orbitrap-IRMS Parameters

We performed stable carbon isotope measurements of PFOA using a Vanquish Neo ultrahigh-performance liquid chromatograph (UHPLC; Thermo Fisher Scientific) coupled to an Orbitrap Exploris 240 mass spectrometer (Thermo Fisher Scientific). We used an OptaMax NG electrospray ionization source with an HESI LOFLO needle insert. Instrument and probe parameters are shown in Table 1. For each analysis, 50 μL of 1.0 mg L^–1^ analyte was introduced into a 25 μL sample loop and injected into the Orbitrap over a 14.5 min window. No chromatographic columns were utilized, allowing for continuous introduction of analyte to the Orbitrap. To prevent sample broadening, we set an initial flow rate of 10 μL min^–1^ for 1 min and then dropped the flow rate to 3 μL min^–1^ to begin sample analyses. Over the course of a sample analysis, we applied a gradient flow rate from 3 μL min^–1^ to 2.75 μL min^–1^. After this, the introduction flow path was changed using a six-port valve, allowing a reference PFOA-01 solution to be injected via a syringe pump at 3 μL min^–1^ from a 1 mL gastight syringe, as shown in Figure 1. Each sample was analyzed in triplicate, followed by a blank injection of 50:50 acetonitrile/methanol and a standard injection of the reference solution (PFOA-01) via UHPLC. Four samples (PFOA-01, PFOA-04, PFOA-13, and PFOA-21) were analyzed across multiple days to assess the long-term measurement precision of the method. Stable carbon isotope ratios were similarly collected for both ESI-generated [PFOA-H]^−1^ ions and [PFOA-CO_2_H]^−1^ fragment ions. Analysis sequences of [PFOA-CO_2_H]^−1^ were performed independently of the [PFOA-H]^−1^ measurements.

**Table 1:** Instrumental Parameters for the Isotope Analysis of Perfluorooctanoic Acid and the Decarboxylated Fragment Ion

	**[PFOA-H]** ^**–1**^	**[PFOA-CO**_**2**_ **H]** ^**–1**^
**Concentration**	1 mg L^–1^	1 mg L^–1^
**Polarity**	Negative	Negative
**Flow rate**	3 μL min^–1^	3 μL min^–1^
**Scan Range**	411–416 (*m*/*z*)	367–372 (*m*/*z*)
**Nominal Resolution at 200** (*m*/*z*)	30,000	30,000
**AGC target**	200%	500%
**Max. Injection Time**	100 ms	100 ms
**Sheath gas**	8 arb. units	10 arb. units
**Aux gas**	5 arb. units	5 arb. units
**Sweep gas**	0 arb. units	0 arb. units
**Spray voltage**	2400 V	2400 V
**RF Lens**	65%	90%

**Figure 1:**
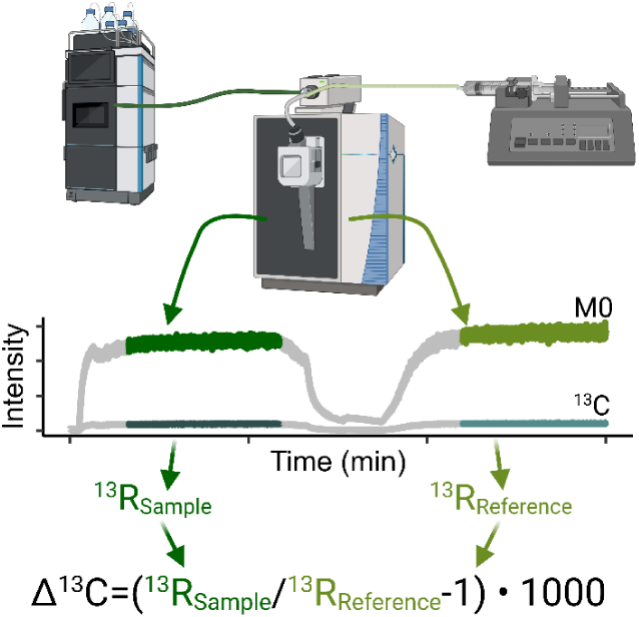
PFAS samples were injected into the mass spectrometer via an ultrahigh-performance liquid chromatograph, followed by a reference solution injected via a syringe pump. The isotope ratios of the sample and reference were calculated from the intensities of the ^13^C-substituted ion to the isotopically unsubstituted ion.

### 2.3 Stable Isotope Data Processing

We collected data using Xcalibur (Thermo Fisher Scientific) software, with resulting .RAW files combined and converted to .Isox files using IsoX (Thermo Fisher Scientific). We used the “isoorbi” package^33^ in R (v 4.3.1)^34^ for extracting ion abundance data from the time frame of analysis. We manually excluded any scans showing a ^13^C isotopically substituted peak but no monoisotopic peak. This isoorbi package also enabled filtering of the data for satellite peaks, low-abundance isotopocules, and scans with the lowest 10% and highest 1% of total ion current (TIC). Isotope ratios of each scan for standards, reference injections, and samples were calculated using

1 RM+1=I[M+1]/I[M0]

where ^*M* +^ ^1^ *R* is the isotope ratio, *I*_[*M* + 1]_ is the intensity of the singly isotopically substituted *M* + 1 peak, and *I*_[*M*0]_ is the intensity of the monoisotopic *M*0 peak. The average of all scans ^*M* + 1^ *R*_ref_ and ^*M* + 1^ *R*_Sample_ for each syringe-injected reference and sample, respectively, were calculated and used to determine Δ^13^C:

2 $$\Delta^{13}C\left(\mathrm{in}‰\right)={(}^{M+1}R_{\mathrm{Sample}}{/}^{M+1}R_{\mathrm{Reference}}-1)\times 1000$$

We calculated the average total ion current (TIC) of all scans for a given sample’s TIC_Sample_ and for its corresponding reference TIC_Reference_. For every sample, the relative TIC was calculated using

3 $$\mathrm{Relative TIC}\;(\mathrm{in}\%)=\left(\mathrm{TI}C_{\mathrm{Sample}}/\mathrm{TI}C_{\mathrm{Reference}}-1\right)\times 100$$

We applied a linearity correction based on relative TIC differences between the sample and reference. Samples were corrected to a value of 0% relative TIC difference between the samples and reference, with a calibration curve based on the isotopic analyses of the standards (see Section 3.4).

### 2.4 Isomeric Analysis

We identified the presence of branched PFOA isomers using a Prominence high-performance liquid chromatography system (HPLC; Shimadzu) coupled to a QTRAP 4500 triple quadrupole-linear ion trap mass spectrometer (SCIEX). A 0.30 mL min^–1^ flow rate carried an injection volume of 4 μL through the HPLC. A C18 column (50 × 2.0 mm, 5 μm particle size, Gemini) separated linear and branched isomers of PFOA, with samples identified by retention times compared to a calibration standard within ±0.1 min. Phase A of the binary mobile phase consisted of LC-MS grade water (Honeywell) containing 20 mM ammonium acetate (Hampton Research), and Phase B consisted of LC-MS grade acetonitrile (Millipore Sigma Supelco). We set a gradient flow to pre-equilibrate the column with 10% Phase B for 2 min, increased to 60% Phase B from 0.5 to 4.0 min, to 90% Phase B from 4.0 to 7.5 min, and held at this ratio until 10.0 min. The mass spectrometer was operated in negative ionization mode, and PFOA was analyzed using scheduled multiple reaction monitoring mode (sMRM), in which dwell time was optimized to enhance sensitivity. The ion spray voltage was set at 4,500  V and temperature at 600 °C. The declustering potential, entrance potential, collision energy, and collision cell exit potential were set at −35, – 10, −14, and −7 V, respectively. Curtain gas pressure, collision gas pressure, and ion source gas pressure were set at 35, 6, and 50 psi, respectively.

## 3 Results and Discussion

### 3.1 Orbitrap-IRMS Measurements of PFOA Isotope Ratios

Our observed sample variations of Δ^13^C values for [PFOA-H]^−1^ are both measurable and reproducible, spanning a range of −23.0‰ to 1.7‰ (Figure 2, Table S.B.1). The average standard deviation across analytical triplicates of all [PFOA-H]^−1^ samples is 0.3‰; however, the long-term average standard deviation of samples with *n* > 3 replicates, measured across multiple days, is 0.7‰. For [PFOA-CO_2_H]^−1^, Δ^13^C values span from −24.6‰ to 3.5‰, with an average standard deviation of 0.2‰, both in the short-term and as measured across several days.

**Figure 2:**
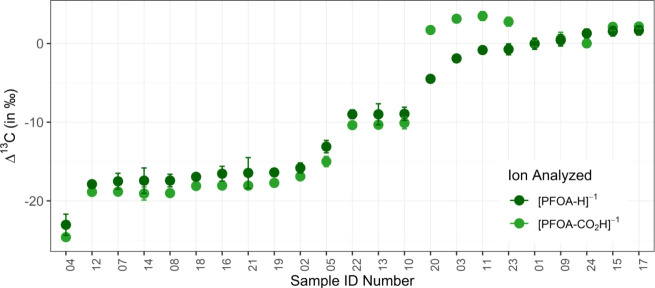
Isotope ratio values of [PFOA-H]^−1^ and its decarboxylated fragment [PFOA-CO_2_H]^−1^ are displayed in dark green and light green, respectively. Error bars show two standard deviations. In points without visible error bars, the point size is larger than two standard deviations.

Similar to the observations of Dombrowski et al.,^23^ our most extreme samples differed by approximately 25‰. We did not identify any apparent correlation between the manufacturer and the measured isotope ratio. Instead, observed variations appeared to be specific to the lot number. For example, the samples supplied by Accustandard (PFOA-02 and PFOA-24), varied by ∼ 17‰ despite sharing the same catalog number, and the samples procured from Aladdin Scientific (PFOA-10 through PFOA-14) showed a wide range of values from approximately −1‰ to −18‰. This phenomenon has been observed previously for other compounds and suggests variations in the source reagents for the synthesis of the final PFAS compounds over time.^35^

As expected, the isotope ratio values of both [PFOA-H]^−1^ and [PFOA-CO_2_H]^−1^ are similar for most samples, reflecting the relatively minor contribution of a single carbon atom from the carboxyl group to the isotope ratio of the entire eight-carbon compound. Despite the similarity between the isotope ratios of PFOA and its fragment ion for a majority of these samples, PFOA-03, PFOA-11, PFOA-20, and PFOA-23 showed large differences between the two, indicating that the carboxyl carbon is significantly more depleted in ^13^C than in the other samples (one-tailed *t*-test, *p* < 0.0005). The differences in isotopic content of [PFOA-H]^−1^ and [PFOA-CO_2_H]^−1^ add an extra dimension to assist in differentiating between PFOA samples. Of the 253 possible pairwise comparisons between samples, 201 samples could be differentiated using Δ^13^C_[PFOA‑H]‑1_ values alone (ANOVA, *p* < 0.05). Using the Δ^13^C_[PFOA‑CO2H]‑1_ values alone, 26 additional pairs could be distinguished (ANOVA, *p* < 0.05). Integrating the two data sets allows three additional samples to be differentiated from one another (Figure 3, Benjamini-Hochberg adj. *p* < 0.05). We need to keep in mind, however, that all 253 pairwise comparisons should not necessarily be differentiable based solely on isotopic value. Though we obtained our samples from multiple different suppliers with different lot numbers, it is possible that the suppliers sourced their PFOA from the same manufacturer.^36^ Regardless of the actual number of comparisons that represent unique samples and sample histories, our method differentiated 91% of the total samples.

**Figure 3:**
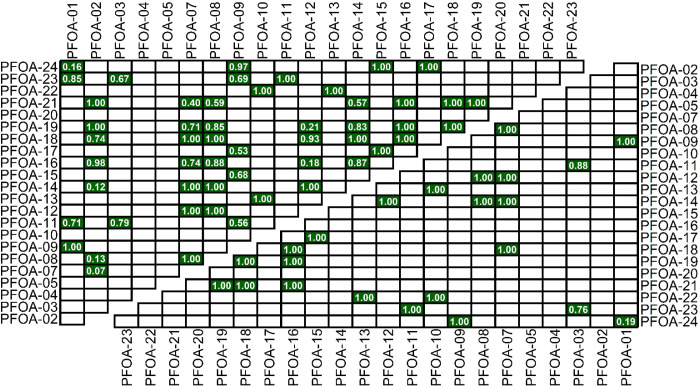
Samples in white are capable of being differentiated from one another (ANOVA, *p* < 0.05), and samples in green are unable to be differentiated. The upper triangle is based on PFOA Δ^13^C data alone; the lower triangle is based on differentiation using the combined PFOA and fragment isotopic values (ANOVA, Benjamini-Hochberg adj-*p* < 0.05). Text reports *p*-values.

### 3.2 Interpretation and Use of Δ^13^C versus δ^13^C

At the maximum resolution available on the Orbitrap Exploris 240 (240,000 fwhm at 200 *m*/*z*), the ^17^O-substituted and ^13^C-substituted peaks could not be resolved. Thus, the isotope ratios observed for the [PFOA-H]^−1^ ion are an integrated measure of both ^13^C and ^17^O, as has been previously described.^37^ Due to the higher abundance of carbon versus oxygen in PFOA and the higher natural abundance of ^13^C (∼1.1%) versus ^17^O (∼0.037%), “Δ^13^C” is used nominally to represent the combined measurement of the ^13^C and ^17^O isotope ratios, and for the [PFOA-CO_2_H]^−1^ isotope ratio measurements that contain no oxygen.

Further, unlike traditional isotopic analyses, carbon isotope ratio values reported here are standardized to an in-house standard (PFOA-01) set to 0‰ and not tied to an internationally recognized standard (e.g., VPDB). As we standardized data to PFOA-01, ad hoc interlaboratory calibration of data generated using this method would be impossible, and any comparison would require a common subset of samples to enable data comparability. However, given the compound-specific and intramolecular nature of Orbitrap-IRMS analyses, standardization of isotope ratios also requires the use of compound-specific and intramolecularly defined standards. For some compounds of interest, there are standards that meet these requirements. Unfortunately, for PFOA, isotopically defined standards are currently not available, especially at the intramolecular scale. To the authors’ awareness, there are no known methods for performing intramolecular isotopic analyses of PFOA that we could perform in-house. Traditional EA-IRMS analysis was attempted for a select number of samples; however, the chemical/thermal stability of these compounds likely caused incomplete conversion of analyte to CO_2_, resulting in poor precision (Table S.C.1). Despite this, isotopic measurements via EA-IRMS and Orbitrap-IRMS of the eight-carbon PFOA showed a strong linear correlation and a consistent difference between the results (Figure S.C.1). Increasing replicates of EA-IRMS measurements heavily risks the degradation of an EA-IRMS system due to the HF produced in combustion. EA-IRMS also would allow for only the calibration of the full [PFOA-H]^−1^ ion to an internationally recognized scale, not the fragment [PFOA-CO_2_H]^−1^ ion. Rather than providing isotopic measurements for these two ions where one is tied to VPDB and one is not, we report all values relative to a single in-house standard for consistency. Additionally, in the context of source attribution, an absolute value on an internationally recognized scale is not necessary for the application space proposed in this study, only relative differences between samples are required. Often, Orbitrap-IRMS methodologies enable measurements that are extremely difficult or infeasible to make on standard isotope ratio equipment. As Orbitrap-IRMS methodologies and applications proliferate, the stable isotope community will need to address optimal approaches for standardization and interlaboratory comparability of measurements made on this emergent platform, especially in cases where it is not possible to make analogous measurements against an isotope standard calibrated to VPDB. This may require the establishment of compound-specific standards through the analysis of multiple samples of the same compound from different sources (as in this study) by multiple Orbitrap-IRMS laboratories.

In many contexts, including an environmental forensic one, only the relative differences between samples are needed for sample differentiation, regardless of the scale on which the isotope ratios are reported. As a result of this approach to scaling, an identical Δ^13^C measure of [PFOA-H]^−1^ and [PFOA-CO_2_H]^−1^ does not necessarily indicate that the isotopic values of the carboxylated and decarboxylated ions, as measured on the VPDB scale, would be the same—only that the relative difference in isotope ratios between the given samples and the reference PFOA-01 are equivalent.

### 3.3 Isomeric Analyses and Potential Synthesis Insights

As synthetic compounds, PFAS are commonly produced from precursors originating from fossil fuel sources. Methane-based sources of carbon are known to have depleted stable isotopic signatures compared to oil/coal-derived carbon.^38^ The combination of isotopic and isomeric signatures in this study indicates that different synthesis methods are associated with different carbon sources. Exclusively linear synthesis of PFOA arises through mechanisms, such as fluorotelomerization, whereas branched isomers are formed through processes such as ECF or LPDF.^18,19^ Isomeric analyses of the PFOA samples used in this study showed that, of the samples where the Δ^13^C_[PFOA‑H]‑1_ was greater than −5‰, seven of the nine contained branched isomers, and the remaining two (PFOA-15 and PFOA-24) were exclusively linear. All samples below the −5‰ threshold demonstrated linear-only isomeric patterns. These results track with prior research indicating that more ^13^C-enriched samples of PFOA tend to show branched patterns, but not exclusively.^23^ However, the broad sample set provided in this study shows that isotopically depleted samples are exclusively linear (Table S.D.1, Figures S.D.1 and S.D.2).

These isotopic results are consistent with known precursors for the different synthesis methods. In ECF and LPDF, an octanoyl halide is reacted with HF or F_2_ to produce PFOA.^39^ Octanoyl halides are often produced from octanoic acid, which is often industrially produced from octanol. Octanol is commonly produced via one of two processes: 1) the hydroformylation and oxidation of petroleum-derived 1-heptene, or 2) through the Ziegler-Alfol process, which reacts four ethenes together in the presence of Al, H_2_, O_2_, and water.^40,41^ In fluorotelomerization, small perfluorinated ethene monomers react to form longer, straight-chain PFAS, where ethene, in turn, is often industrially produced from Fischer–Tropsch reactions with primarily natural gas-derived carbon monoxide.^32,42^ The enriched isotopic signatures indicate that manufacturers utilizing branched-isomer-producing PFOA synthesis processes favor the use of the longer-chain petroleum-derived precursors, whereas the isotopic depletion of the linear isomers indicates that manufacturers using fluorotelomerization often source their precursor materials from methane-derived carbon (Figure 4, Table S.C.1). The two PFOA samples with enriched isotopic signatures and exclusively linear isomers could indicate that carbon monoxide used in forming the perfluorinated ethenes originates from incomplete combustion of petroleum or coal.

**Figure 4:**
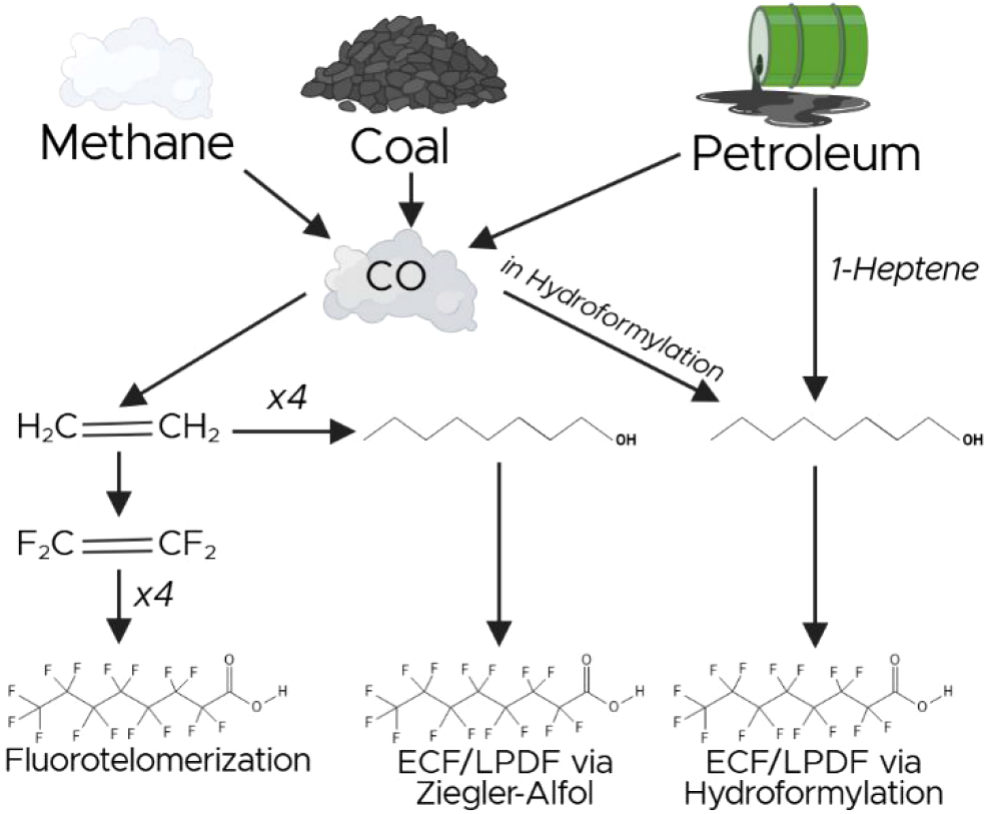
A schematic shows the potential movement of carbon through the synthesis process. Arrows can indicate multiple reaction steps, with prominent intermediates shown. The isotopic signature of a product is reflective of the source material, where methane is typically more depleted in ^13^C compared to oil/coal sources.

Fragments of samples containing branched isomers showed significant enrichment compared to fragments of samples containing only linear forms of PFOA (one-tailed *t*-test, *p* = 0.002, Figure S.D.3). The Δ^13^C_[PFOA‑CO2H]‑1_ values of samples containing only linear isomers were, on average, 1.2‰ lighter than the Δ^13^C_[PFOA‑H]‑_, while the Δ^13^C_[PFOA‑CO2H]‑1_ values of the samples containing branched isomers were, on average, 2.8‰ heavier than the corresponding Δ^13^C_[PFOA‑H]‑1_. Among the branched isomer samples, two subgroups of samples showed significant differences (one-tailed *t*-test, *p* = 0.001): a “high enrichment” set (PFOA-03, PFOA-11, PFOA-20, and PFOA-23) with a 3.6 to 5.9‰ enrichment upon decarboxylation, and “no/slight enrichment” (PFOA-01, PFOA-07, PFOA-09, and PFOA-15) with a 0 to 0.6‰ enrichment upon decarboxylation.

In the cases with observed isotopic enrichment in the associated decarboxylated fragment compared to the full PFOA ion, the carboxyl carbon is more depleted in ^13^C than the carboxyl group of standard PFOA-01. Due to the Δ^13^C values of both [PFOA-H]^−1^ and [PFOA-CO_2_H]^−1^ being corrected on different scales, direct calculation of the Δ^13^C of the carboxyl carbon is not possible. Still, the full [PFOA-H]^−1^ isotopic signature is the weighted average of all eight carbons, and thus big differences between the Δ^13^C of [PFOA-H]^−1^ and [PFOA-CO_2_H]^−1^ upon the loss of the single carboxyl carbon may indicate an extreme isotopic depletion of this carbon. Thus, the “high enrichment” set of samples noted earlier may be indicative of the hydroformylation synthesis pathway using methane-derived CO when reacted with petroleum-derived 1-heptene.

Only one sample (PFOA-15), as indicated by LC-MS/MS analysis to contain exclusively linear isomers, showed the petroleum isotopic signature and no/slight enrichment with decarboxylation. As all other linear isomeric samples show depletion instead of enrichment, it is possible that PFOA-15 was produced using LPDF or ECF and contains branched isomers that were unresolvable in our LC-MS/MS analysis. Alternatively, the purification process utilized by the supplier may be highly effective at the removal of branched isomers, resulting in seemingly contradictory isotopic and isomeric signatures.

In a source attributional context, the binary indicator of the presence/absence of branched isomers allows for the further differentiation of three additional pairwise comparisons: PFOA-15 was from PFOA-17, PFOA-24 was from PFOA-01, and PFOA-24 was from PFOA-09. This isomeric fingerprint may also be encoded in the isotopic information provided by the offset in isotopic values between [PFOA-H]^−1^ and [PFOA-CO_2_H]^−1^. Though not a completely orthogonal dimension to the information provided by the stable isotopic content, the combination of isotopic information and synthesis indicators of a sample increases the forensic resolving power.

### 3.4 Implications and Limitations

When performing intramolecular measurements via Orbitrap-IRMS, others have utilized a collision cell to measure the fragment isotope ratios.^37,43,44^ However, the yield of [PFOA-CO_2_H]^−1^ ions produced when performing higher-energy collisional dissociation (HCD) cell fragmentation of [PFOA-H]^−1^ ions selected by quadrupole transmission was lower than the throughput of [PFOA-CO_2_H]^−1^ ions formed via fragmentation at the source. Given the high purity of the samples obtained and the absence of higher-order perfluoroalkyl chains in the samples, the in-source fragmentation method can provide greater understanding of the isotopic composition of PFOA in this case. In mixtures of multiple perfluoroalkyl chains, commonly seen in environmental samples, either 1) chemical/chromatographic separations would need to be applied to ensure the parent ion of the measured fragment is PFOA and not a different compound, or 2) mass filtering via quadrupole and subsequent fragmentation in a collision cell would need to be performed with samples at higher concentrations than those presented in this study.

One future avenue that may enable greater forensic discrimination potential is the measurement of isomer-specific isotope ratios. Branched and linear PFOA isomers are synthesized in the same lots with the same precursor reagents; however, the formation of the two different isomeric products may result in different isotopic signatures in each, which are obscured by measuring the two together, as in this study. If the difference in isotopic value is of sufficient magnitude, this could provide an additional dimension of intrinsic signature to these chemically identical samples. Given the varying chemical and physical properties observed between structural isomers, it is possible that differing mechanisms for ionization and fragmentation between the two isomers in the ESI source^45^ could result in differences in the measured isotopic values for isotopically identical samples. Though our data indicate that synthesis processes may impart an isotopic offset between the carboxylated and decarboxylated versions of a given sample, the variations in the isotopic measurement may be due to the varying properties of the different isomers rather than true isotopic differences. If so, regardless of whether differing values of this offset are analytical artifacts from the presence of multiple isomers or representative of true isotopic content, this method still provides an environmental forensic tool capable of differentiating between different samples based on their synthesis pathways. Further, the major differences in isotopic content recorded here are not due to isomeric interference, as evidenced by the isotopic measures of the samples measured using exclusively linear isomer content, with isotopic ratios spanning 24.6‰ and 26.8‰ for [PFOA-H]^−1^ and [PFOA-CO_2_H]^−1^, respectively. To determine the extent to which the isomeric content may impact isotope ratio measurements performed on Orbitrap-IRMS, chromatographic separations of samples to separate the different isomeric forms could enable fraction collection of the different isomers, allowing measurements of the different isomers to be made independently from one another.

One significant limitation of this method is the need for matrix matching between samples and references. As others have previously discussed,^46,47^ Orbitrap-IRMS measurements can be influenced by absolute isotopologue abundance and space charge effects within the quadrupole and the Orbitrap mass analyzer itself. In the application to environmental samples, extensive sample preparation processes will need to be performed to reduce ion suppression in the source, remove unwanted spectral interferences, and normalize concentrations. Some of our data suggest that changes in measured Δ^13^C values due to relative TIC effects are linearly related, implying that a linearity correction may improve the precision and accuracy of the method (Figure 5). The PFOA-01 standard injected through the UHPLC was the same as the reference solution infused via syringe injection, and by definition, the Δ^13^C measured for the standard should be 0‰. This was observed to be true for samples with low relative TIC variations; however, deviations in Δ^13^C values occur at a rate of approximately 0.1‰ per percent relative TIC variation. Applying the linearity correction, we see an improvement in the accuracy of the results from sample PFOA-01, with isotopic values of PFOA-01 improving from an uncorrected value of −1.0‰ to a corrected value of 0.0‰. We also see significant improvements in precision for samples with high variability in measured TIC, with the standard deviation of PFOA-21 improving from 0.9‰ to 0.2‰. Due to the varying concentrations of PFOA in environmental samples, and by extension absolute isotopologue abundance, a linearity correction may be critical to enabling sample comparison.

**Figure 5:**
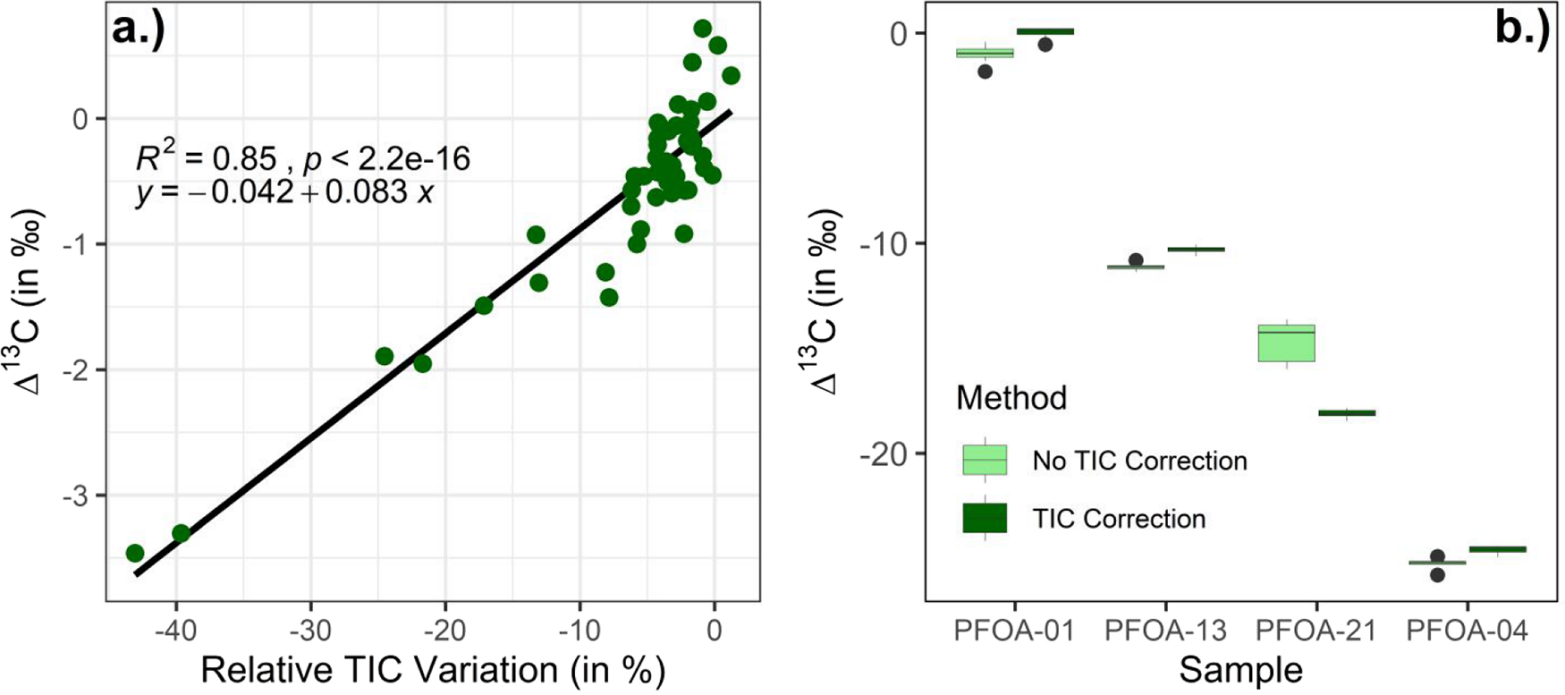
a) Relative TIC variation of each standard replicate was plotted against the measured Δ^13^C values of [PFOA-CO_2_H]^−1^. A linear regression trend is shown in black. b) A linearity correction was applied based on the linear trend in a), normalizing values to a 0% relative TIC variation. Samples measured across multiple days had a wider range of relative TIC variation than samples run on a single day and are plotted before and after the TIC-based linearity correction was applied.

## 4 Conclusion

Our method utilizes the Orbitrap-IRMS method for the stable isotopic analysis of carbon in PFAS compounds in environmentally relevant quantities. Isotopic characterization of PFOA fragments allows for measurements of intramolecular stable carbon isotope ratios, increasing forensic resolving power. Future work should evaluate the impact of multi-isotopic measurements on source attribution potential—incorporating oxygen isotope ratio measurements in addition to carbon to better distinguish between samples. Further, continuing to develop analyses for smaller fragments of PFOA to better understand the intramolecular isotopic composition of the compound would allow for improved differentiation between different sources. Given the vast array of PFAS compounds currently dispersed throughout the environment, extending Orbitrap-IRMS measurements to other congeners would allow for broader applicability of this method to sites not containing PFOA and improved resolution of PFAS sources from environmental samples. An Orbitrap-IRMS analysis of PFOA consumes ∼50 ng of PFOA per analysis. Median concentrations of PFOA in contaminated soils are between 38 and 83 ng g^–1^,^48^ which would enable an Orbitrap-IRMS analysis via extraction of PFOA from 1 g of soil. In drinking water, where the current EPA maximum contaminant limits (MCL) are set to 4 ng L^–1^ for PFOA,^49^ a sample of water at this level or higher would require 12.5 L to isolate enough PFOA for an Orbitrap-IRMS analysis (assuming 100% extraction recovery). This demonstrates a feasible approach compared to EA-IRMS, which would require complete extraction of PFOA from 12,000 to 26,000 g of soil for one replicate analysis or 250,000 L of contaminated water with PFOA at 4 ng L^–1^ MCL. This Orbitrap-IRMS method lays a foundation for extending current stable isotopic methodologies of environmental contaminants—such as for source attribution and remediation monitoring—to PFAS compounds.

## Supplementary Material



## Notes

Data tables and figures containing sample information, isotopic values of Orbitrap-IRMS measured ratios, EA-IRMS data, and isomeric presence and absence (PDF)

## Abbreviations

PFAS: Per- and polyfluorinated alkyl substances
ECF: electrochemical fluorination
LPDF: liquid phase direct fluorination
ESI: electrospray ionization
PFOA: perfluorooctanoic acid
IRMS: isotope ratio mass spectrometry
SNIF-NMR: site-specific natural isotope fractionation nuclear magnetic resonance
UHPLC: ultrahigh-performance liquid chromatograph
HPLC: high-performance liquid chromatograph
TIC: total ion current
MCL: maximum contaminant limits
