# Supplementary material for: Source Fingerprinting of PFOA via Full- and Intramolecular Stable Isotope Ratios of Carbon Using Orbitrap-IRMS
Source: Anal Chem. 2025 Dec 13;97(50):28028–36. doi: 10.1021/acs.analchem.5c06168 (PMC12750406; doi:10.1021/acs.analchem.5c06168)
Supplement: Supplementary file 1 [file ac5c06168_si_001.pdf]

SUPPLEMENTAL INFORMATION

Source Fingerprinting of PFOA via Full- and Intra-molecular Stable Isotope Ratios of Carbon using Orbitrap-IRMS

Holden M. Nelson[1,2,3], Zhiliang Xu[2,4], Hui Li[2,4], and James J. Moran[1,2,4,5]*

[1] *Department of Integrative Biology, Michigan State University, East Lansing, MI 48824*

[2] *Center for PFAS Research, Michigan State University, East Lansing, MI 48824*

[3] *Department of Chemistry, Michigan State University, East Lansing, MI 48824*

[4] *Department of Plant, Soil, and Microbial Sciences, Michigan State University, East Lansing, MI 48824*

[5] *Ecology, Evolution, and Behavior Program, Michigan State University, East Lansing, MI 48824*

**Corresponding Email: moranja7@msu.edu*

TABLE OF CONTENTS

SECTION S.A.— Sample Information………………………………………………………………………..…………S3
SECTION S.B.— Orbitrap-IRMS Results……………………………………………………...………………………...S4
SECTION S.C.— Isomeric Analysis…………………………………………………………………...…………………...S5
SECTION S.D.— EA-IRMS Measurement…………...………………………………………………………………….S7

SECTION S.A. Sample information

Table S.A.1. Sample information for neat stocks of perfluorooctanoic acid (PFOA) obtained from various suppliers.

Sample ID	Acid/Salt	Manufacturer	Lot Number	Purity (%)
PFOA-01	Acid	Sigma-Aldrich	07420DH	96
PFOA-02	Acid	Accustandard	32312	100
PFOA-03	Acid	Acros Organics	A0448212	96
PFOA-04	Acid	Sigma	WXBD5139	95
PFOA-05	Acid	Sigma–Trace cert	BCCD3863	96
PFOA-07	Acid	Ivy Chemical	P230814	97
PFOA-08	Acid	Ivy Chemical	HP240322	98
PFOA-09	Acid	Acros Organics	A0445037	96
PFOA-10	Acid	Aladdin Scientific	I2227683	96
PFOA-11	Na-Salt	Aladdin Scientific	L2118337	97
PFOA-12	NH_4-Salt	Aladdin Scientific	b2328923	98
PFOA-13	Acid	Aladdin Scientific	A2416672	98
PFOA-14	Acid	Aladdin Scientific	I2312597	90
PFOA-15	Acid	Cayman Chemical	0656890-10	100
PFOA-16	Acid	AstaTech	P102-32569	95
PFOA-17	Acid	Matrix Scientific	P13W	98
PFOA-18	Acid	Strem Chemical	L00812307	98
PFOA-19	Acid	Accela ChemBio	R23053236	98
PFOA-20	NH_4-Salt	Chemodex	X9078	---
PFOA-21	Acid	Synquest Laboratories	22778	100
PFOA-22	Acid	Santa Cruz Biotech	E1624	100
PFOA-23	Acid	Aldrich Chemistry	MKBJ5884V	96
PFOA-24	Acid	Accustandard	33202	100

SECTION S.B. Orbitrap-IRMS measurements

Table S.B.1. Results of Orbitrap-IRMS analysis of both $[PFOA-H]^{-1}$ and $[PFOA-CO_2H]^{-1}$ as measured at 1.0 mg L^{-1}.

	$[PFOA-H]^{-1}$			$[PFOA-CO_2H]^{-1}$		
Sample ID	n	$\Delta^{13}C$ (in ‰)	Standard Deviation (in ‰)	n	$\Delta^{13}C$ (in ‰)	Standard Deviation (in ‰)
PFOA-01	13	0.0	0.4	9	0.0	0.2
PFOA-02	3	-15.9	0.3	3	-16.9	0.2
PFOA-03	3	-2.0	0.1	3	3.1	0.3
PFOA-04	9	-23.0	0.7	6	-24.6	0.2
PFOA-05	3	-13.1	0.4	3	-15.0	0.3
PFOA-07	3	-17.6	0.5	3	-18.8	0.1
PFOA-08	3	-17.4	0.4	3	-19.0	0.1
PFOA-09	3	0.3	0.4	3	0.6	0.4
PFOA-10	3	-9.0	0.4	3	-10.1	0.4
PFOA-11	3	-0.8	0.1	3	3.5	0.3
PFOA-12	3	-17.9	0.3	3	-18.9	0.0
PFOA-13	9	-9.0	0.7	6	-10.3	0.2
PFOA-14	3	-17.4	0.8	3	-19.1	0.4
PFOA-15	3	1.6	0.3	3	2.1	0.2
PFOA-16	6	-16.6	0.5	3	-18.1	0.2
PFOA-17	3	1.6	0.3	3	2.2	0.1
PFOA-18	3	-17.0	0.2	3	-18.1	0.1
PFOA-19	3	-16.4	0.3	3	-17.7	0.3
PFOA-20	6	-4.2	1.0	3	1.7	0.2
PFOA-21	9	-16.3	1.0	9	-18.1	0.2
PFOA-22	3	-9.1	0.3	3	-10.4	0.2
PFOA-23	3	-0.8	0.4	3	2.8	0.3
PFOA-24	3	1.2	0.3	3	0.0	0.3

Section S.C. Elemental analyzer isotope ratio mass spectrometer (EA-IRMS) Measurement

Methodology

EA-IRMS Measurement

Approximately 1 mg of neat powdered PFOA-01, PFOA-04, PFOA-11, and PFOA-21 was weighed and wrapped into a silver capsule. The silver capsule was subsequently wrapped inside of a tin capsule. The tin capsule was dropped into a Isotope Cube elemental analyzer (Elementar) where a CuO combustion reactor oxidized the sample at 950°C. Helium carried the resultant gas at a flowrate of 229 mL min^{-1} through a copper reduction reactor at 650°C and a phosphorous pentoxide water trap to remove oxygen gas and water, respectively. Isotope ratios of the CO_2 gas were measured on an Isoprime VisION (Elementar). Samples were calibrated to Vienna Pee Dee Belemnite (VPDB) using in-house standards of known isotopic value: wheat ($\delta^{13}C_{VPDB}$ = -25.55 ‰) and sorghum ($\delta^{13}C_{VPDB}$ = -13.67 ‰).

To place the EA-IRMS isotopic measurements on the same scale as those of the $[PFOA\text{-}H]^{-1}$ and $[PFOA\text{-}CO_2]^{-1}$, a $\Delta^{13}C$ value was calculated relative to PFOA-01 such that

$$\Delta^{13}C\ (in\ ‰) = [\delta^{13}C_{VPDB}]_i - [\delta^{13}C_{VPDB}]_{PFOA-01} \qquad \text{eq S.1.}$$

where *i* denotes the Sample ID.

Table S.C.1. Results of EA-IRMS samples of four neat PFOA samples.

Sample ID	n	$\delta^{13}C_{VPDB}$ (in ‰)	$\Delta^{13}C$ (in ‰)	Standard Deviation (in ‰)
PFOA-01	5	-28.1	0.00	0.8
PFOA-04	5	-52.0	-24.0	1.6
PFOA-11	4	-29.0	-0.9	1.0
PFOA-21	4	-47.2	-19.1	0.1

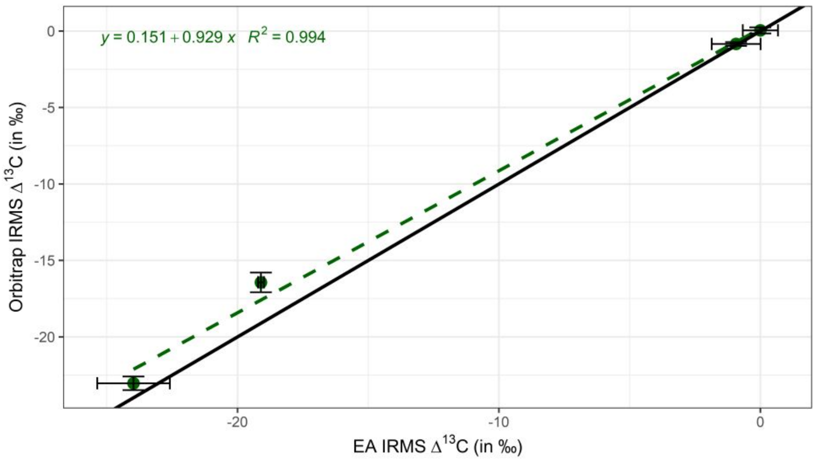

Figure S.C.1. Four samples of PFOA were analyzed via both EA-IRMS and Orbitrap-IRMS. Black bars indicate 95% confidence intervals. The green equation and dashed line are fit to the data, while the solid black line represents the identity.

As seen in Figure S.C.1. the Orbitrap-IRMS and EA-IRMS data are linearly correlated, however, there appears to be a scale compression effect between the two methods. The exact cause of this deviation is difficult to determine. Much as in other work using EA-IRMS to examine isotopic values of PFOA,[1] due to the relatively extreme isotopic values of the most ^{13}C depleted samples analyzed ($\delta^{13}C_{VPDB}$ = -52‰), we were unable to bracket the low end of the range with isotopic standards. Thus, the observed compressive effect may be due to error in the measurement by the EA-IRMS rather than or in addition to error from the Orbitrap-IRMS measurement. Further, we attribute the variable and poor precision of EA-IRMS measurements of PFOA in Table S.3.1 to sample resistance to oxidation. The long-term standard deviation of both in-house EA-IRMS standards is 0.2‰.

Section S.D. Isomeric Content Results

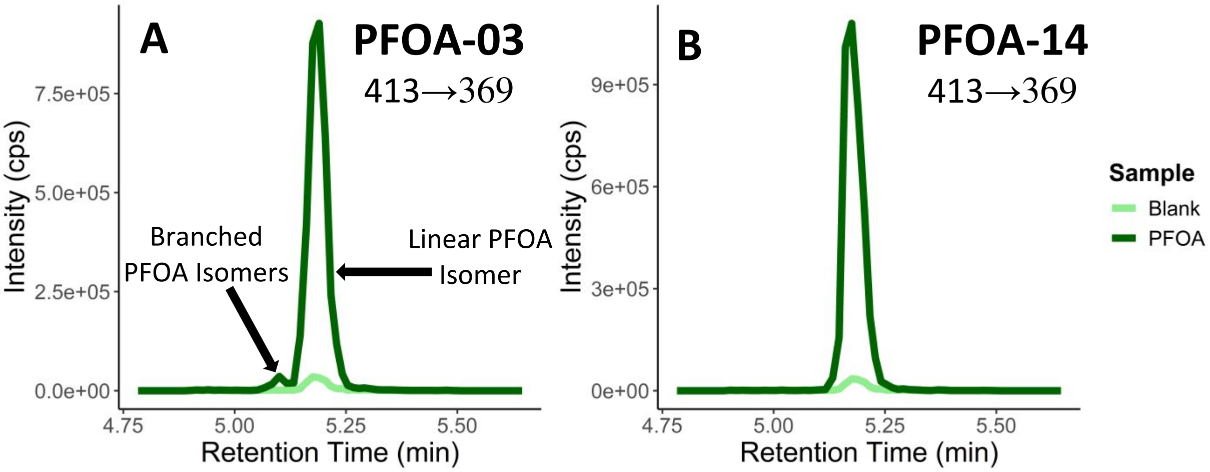

Figure S.D.1. Selected chromatograms from the isomeric analysis of PFOA are shown with intensities from the first transition ion 413 m/z → 369 m/z. In A.), PFOA-03 was determined to have branched PFOA isomers present due to the elevated intensity peak eluting before (5.10 min) the linear isomer PFOA peak (5.17 min). In B.), PFOA-14 was determined to have no branched PFOA isomers present due to the absence of the peak at 5.10 minutes.

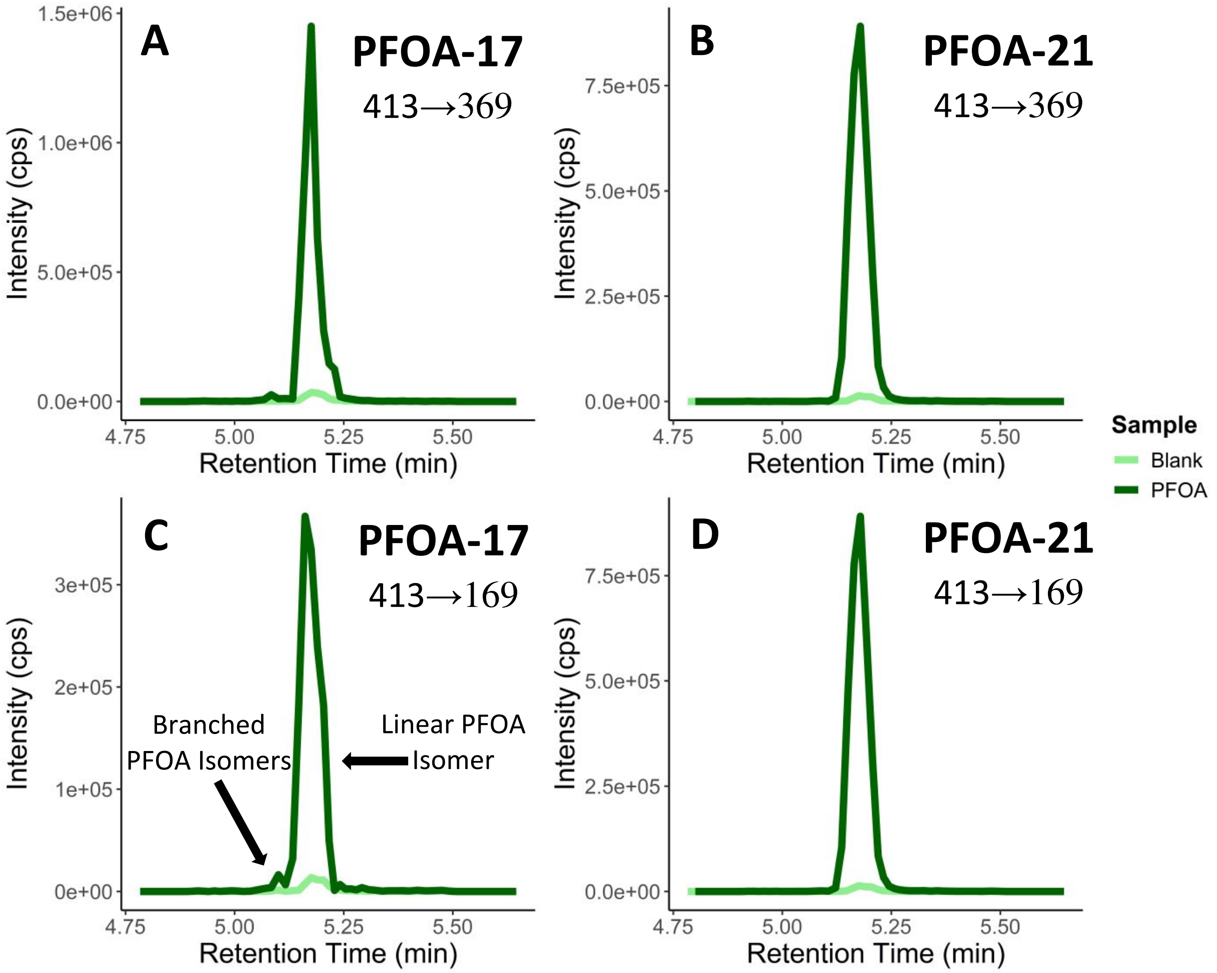

Figure S.D.2. Selected chromatograms from the isomeric analysis of PFOA are shown with intensities from both the first transition ion 413 m/z → 369 m/z and the second transition ion 413 m/z → 169 m/z. In A.), the presence of branched isomers in PFOA-17 was indeterminate based on the low intensity of the peak at 5.10 min. In B.), PFOA-21 was determined to have no branched PFOA isomers present due to the absence of the peak at 5.10 minutes. In C.), the second transition ion shows heightened sensitivity to isomer presence, and thus was used as a diagnostic tool to identify presence/absence of branched PFOA isomers in addition to the first transition chromatogram. In D.), the second transition chromatogram shows no peak at 5.10 min, as in B.), and providing further assurance no branched isomers are present in PFOA-21.

Table S.D.1. Comparing the difference in $\Delta^{13}C$ values of the bulk and decarboxylated PFOA fragment $[PFOA\text{-}H]^{-1}$ $\Delta^{13}C$ – $[PFOA\text{-}CO_2H]$ $\Delta^{13}C$ and the presence/absence of branched isomers.

**Sample ID**	**$\Delta^{13}C$ Difference**	**Branched Isomers Present?**
PFOA-20	5.9	Y
PFOA-03	5.1	Y
PFOA-11	4.3	Y
PFOA-23	3.6	Y
PFOA-17	0.6	Y
PFOA-15	0.6	N
PFOA-09	0.3	Y
PFOA-01	0.0	Y
PFOA-02	-1.0	N
PFOA-12	-1.0	N
PFOA-10	-1.1	N
PFOA-24	-1.1	N
PFOA-18	-1.2	N
PFOA-07	-1.3	N
PFOA-22	-1.3	N
PFOA-19	-1.3	N
PFOA-13	-1.4	N
PFOA-16	-1.5	N
PFOA-08	-1.6	N
PFOA-04	-1.6	N
PFOA-14	-1.6	N
PFOA-21	-1.7	N
PFOA-05	-1.9	N

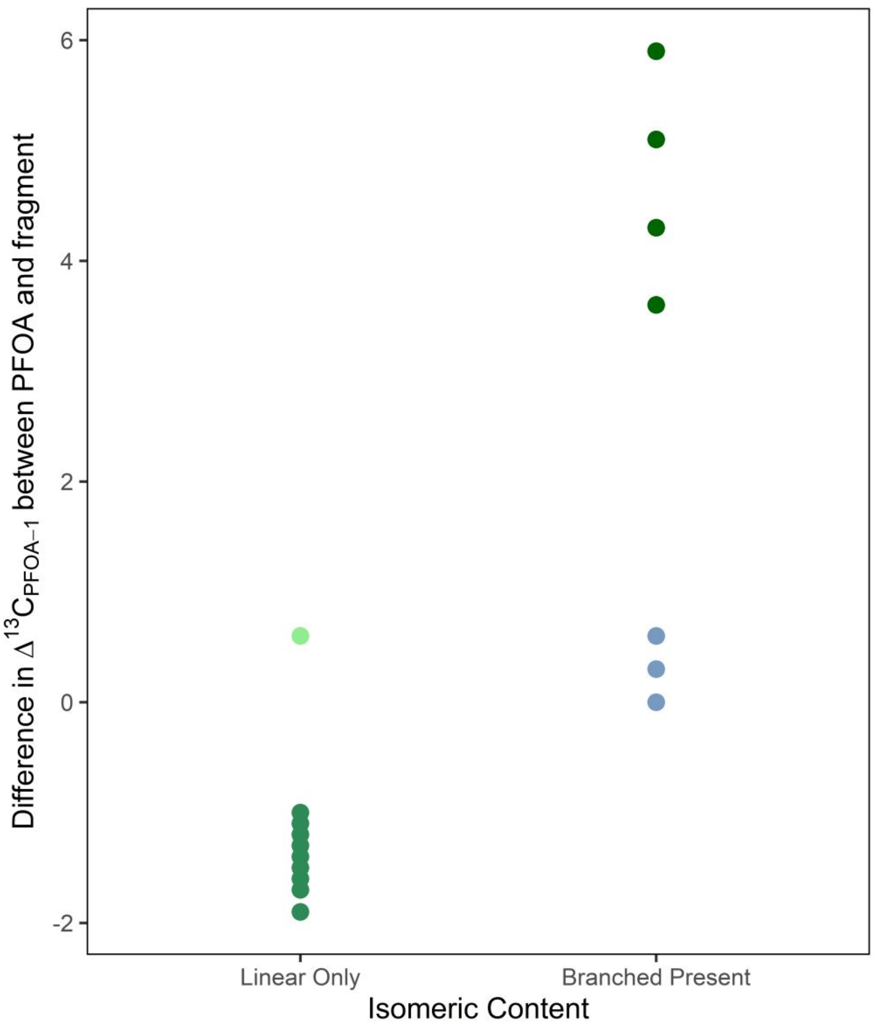

Figure S.D.3. Comparing the difference in $\Delta^{13}C$ values of the bulk and decarboxylated PFOA fragment $\Delta^{13}C_{[PFOA\text{-}H]\text{-}1}$ – $\Delta^{13}C_{[PFOA\text{-}CO2H]\text{-}1}$ and the presence/absence of branched isomers. The different colors are indicative of statistically significant groups of samples with the light green representing PFOA-15.

References

(1) Dombrowski, A.; Wojtal, P. K.; Pan, H.; Lane, C. S.; Mead, R. N. Stable Carbon and Sulfur Isotopic Compositions of Per- and Polyfluoroalkyl Substances. *Environ. Sci. Technol. Lett.* 2025, *12* (2), 216–221. https://doi.org/10.1021/acs.estlett.5c00021.
